# The Double-Edged Effects of Dual-Identity on the Emotional Exhaustion of Migrant Workers: An Existential Approach

**DOI:** 10.3389/fpsyg.2020.01266

**Published:** 2020-06-10

**Authors:** Xiaobei Li, Hongyu Zhang, Jianjun Zhang

**Affiliations:** ^1^Graduate School of China, Sungkyunkwan University, Seoul, South Korea; ^2^CUFE Business School, Central University of Finance and Economics, Beijing, China; ^3^Guanghua School of Management, Peking University, Beijing, China

**Keywords:** emotional exhaustion, migrant workers, identity, work perceptions, HRM strength

## Abstract

By integrating an existential approach to burnout, identity theory, and the job demand–resource (JD–R) model, this paper compares the sense-making processes of migrant workers who embrace both rural and urban identities (i.e., dual-identity holders) with those who suppress either identity (i.e., non-dual-identity holders). In particular, we have examined these dual-identity holders’ interpretations of the workplace regarding internal corporate social responsibilities (CSR) efforts and job complexity and the subsequent emotional exhaustion. A sample of 1,985 migrant workers in China reveals that dual-identity holders may have decreased emotional exhaustion because of higher perceptions of internal CSR efforts, and increased emotional exhaustion because of higher perceptions of job complexity. Furthermore, it is found that human resource management (HRM) strength (i.e., employees’ shared perceptions of HR practices) weakens those two relationships. These findings have important implications for managing migrant workers and ensuring their well-beings.

## Introduction

As the world becomes increasingly interconnected, workforces have become more mobile (Choi et al., 2017; Huff et al., 2017). Although migrant workers add diversity that enhances organizational learning, creativity, and problem-solving (Cox et al., 1991; Ely and Thomas, 2001) the lack of research into their human resource management (HRM) is palpable (Al Ariss and Sidani, 2016). When compared with the locals, migrant workers are sometimes treated unfairly, resulting in decreased job satisfaction and lower commitment (Syed, 2008). Thus, to obtain sustainable output from migrant workers, more research into understanding their work experiences and existential needs is imperative.

In this study, we attempt to understand migrant workers from the identity perspective because it is identity that defines who a person is and fundamentally influences perceptions and thoughts. A key characteristic difference between migrant workers and local workers involves their identities regarding the sense of belonging to a place (Zhang et al., 2019). After migration, migrant workers can adapt their identities to two possible places: home and host communities. Thus, they consistently face issues of identity reformation; that is to say, their identity remains in a state of flux (Berry, 1997). As documented in several lines of literature (i.e., social identity complexity and biculturalism), similar to bi-culturalism, some migrant workers develop a unique identity that is connected to both home and host communities. We call this group of migrant workers *dual-identity holders*. These works in the literature have been found to perceive fewer threats to their self-concept, higher interpersonal tolerance, and higher dialectical self-belief (Roccas and Brewer, 2002; Huff et al., 2017; Zhang et al., 2017). However, with few studies having examined migrant workers’ feelings about the workplace, we aim to explore whether dual-identity holders perceive higher or lower emotional exhaustion in the workplace and seek to understand the mechanisms.

*Emotional exhaustion* is the core component of burnout (Grossi et al., 2003; Bakker et al., 2004). Burnout is known as “a psychological syndrome emerging as prolonged response to chronic interpersonal stressors on the job,” including emotional exhaustion, depersonalized cynicism, and diminished personal accomplishment (Maslach and Leiter, 2016, p. 103). Emotional exhaustion differs from the other two components (Kristensen et al., 2005; Hu and Schaufeli, 2011) because emotions dealing with personal mental resources are evaluative responses to environmental stimuli (Frijda, 1988) whereas the rest are consequences of exhaustion.

Our framework is based on the existential approach of burnout (Pines, 1993) which asserts that emotional exhaustion is a consequence of dealing with uncertainty. When migrant workers move to a new environment, they experience discrepancies between reality and the ideal-self/life. Such discrepancies or disappointments deplete one’s personal mental resources. Integrating this approach with the job demand–resource model (JD–R) and social identity complexity (SIC) theory, we propose and test two pathways that lead to emotional exhaustion among migrant workers. First, in relation to job-resource, dual-identity holders who have the fortitude to tolerate organizational challenges and trust themselves to deliver complex work products in order to attain their goals view their organizations in a more positive light and then have lower emotional exhaustion. We empirically tested this idea by using the construct “internal CSR efforts” which refers to practices that care about the welfare of internal workforce (i.e., employees) (Hameed et al., 2016). Internal CSR has relatively broader meanings (Farooq et al., 2017) and we in this paper operationalized it based on the content of SA8000 which is an international certification standard that helps protect human rights in the workplace (Li et al., 2012). Second, in relation to job demand, dual-identity holders who engage in more workplace sense-making will perceive higher job complexity and then experience higher emotional exhaustion.

**FIGURE 1:**
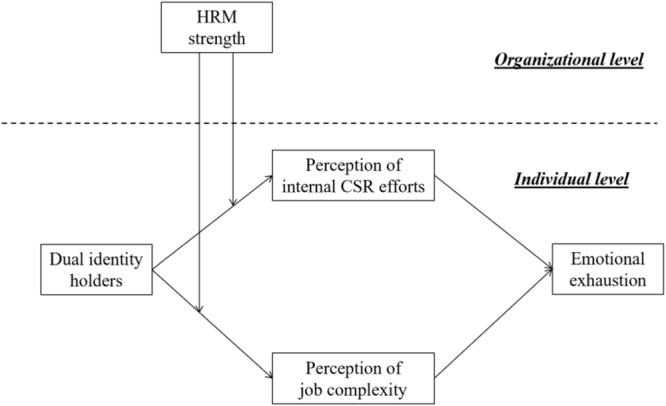
Hypothesized model.

Our second objective is to examine the moderating effects of HRM strength, which is an organizational boundary condition that limits the differences between dual-identity holders and non-dual-identity holders. *HRM strength* is defined as employees’ shared perceptions of the organizational HRM system (Bowen and Ostroff, 2004; Ostroff and Bowen, 2016). Organizations with high HRM strength can be seen to be more clear-cut and consistent in HR implementation, thereby creating more predictability rather than uncertainty in workplaces. HRM strength is expected to mitigate the differences between dual-identity holders and others. Our research framework is summarized in Figure 1.

The tensions of overlapping and conflicting identities must be handled within larger social or institutional contexts (Barker, 2017; Huff et al., 2017). To test the research model in this paper, we analyzed a large sample of migrant workers in China, which is institutionally divided into rural and urban regions. Many people migrate from rural to urban areas to pursue better lives. Accordingly, dual-identity holders in this research stand for migrant workers who simultaneously identify with the urban and the rural. China is a suitable context in which to test the theoretical model, but it is not a China-specific theoretical model. Our findings should illuminate the work experience of migrant workforces in general.

This paper makes three contributions. First, we integrate the literature regarding multiple identities and the existential approach to burnout to test a detailed path model focusing on migrant workers’ identities and work perceptions. We demonstrate that migrant workers vary their work states with differing perceptions regarding their identities. Holding dual identities can be both beneficial and detrimental. Although dual-identity holders may have more positive personal perspectives, their diffused efforts may render them more susceptible to burnout. Second, we contribute to the emotional exhaustion literature by incorporating the identity perspective. Employee interpretation of organizational ethics and jobs is known to influence resources and subsequent exhaustion (Schwepker and Hartline, 2005; Mulki et al., 2008) and we extend this line of research by shedding light on how those interpretations are related to the self-concepts of migrant workers. Third, we contribute to the HRM literature by further demonstrating that HRM strength influences an organization by affecting migrant workers work perceptions and emotions. These findings provide practical implications for organizational policymakers to better harness workforce diversity.

### Theory and Hypothesis Development

#### Dual-Identity Holders

According to social identity complexity (SIC) theory (Roccas and Brewer, 2002) migrant workers can be conceptualized as high SIC individuals. People often engage with multiple groups associated with various characteristics, beliefs, values, resources, and rituals (Roccas and Amit, 2011). When moving to a new place individuals may maintain their original identities by sustaining contact with associates and families in their home community, and at the same time develop a new identity by creating contacts with new friends and colleagues in the host community (Berry et al., 2006, p. 72). The process of acculturation enables individuals to understand the cultures of each group, while changing the original cultural patterns of both. SIC theory (Roccas and Brewer, 2002) posits that the reconciliation process may cause psychological dissonance, and the result depends on whether individuals are aware of the identity differences and the methods by which to integrate them. High-SIC individuals can recognize and/or reconcile the inconsistencies among identities through high open-mindedness (Hong, 2010) by emphasizing overall value priorities (Roccas and Brewer, 2002) and by developing dialectical self-beliefs (Zhang et al., 2017). Dual-identity holders are those who are able to construct perceptions of different communities such that potentially competing and inconsistent definitions of the social self are reconciled. In contrast, non-dual-identity holders often suppress either home or host community identity to maintain cognitive consistency. They adhere to a singular description of the self and subjectively deny or suppress certain identities to avoid internal conflicts and disconnections (Gil et al., 1994). These people have the tendency to identify with a single group, which they perceive to be superior, exalted, and more favorable to their self-concepts (Zimnermann et al., 2003) or as more commonly represented in their own contexts (Croucher, 2011).

#### Contextualizing Dual-Identity Holders Among Chinese Migrant Workers

Migrants in China comprise a large population that usually moves from rural to urban regions. These two regions are institutionally and culturally different and represent contrasting values and lifestyles. Furthermore, they compete for public resources. The conflicts are largely ascribed to the *hukou* (household registration) system, which grants urban *hukou* social status and privileges for job hunting, education, and medical care (Démurger et al., 2009). This is in stark contrast to the plight of the rural *hukou*, which are usually confined to arduous and unsafe jobs that come with long working hours and low pay (Zhang et al., 2010). Despite recent government reforms, rural citizens find it difficult to acquire urban *hukou* registration. In the organizational context, migrant workers may be mistreated according to job type, salary level, and interpersonal interaction (Frenkel and Yu, 2015). Indigenous sociology studies have demonstrated that urban and rural regions have widened social distances, thus increasing the psychological divide (Guo and Chu, 2004; Lei, 2012).

Despite conflicts between rural and urban regions, Chinese migrant workers may integrate rural and urban identity if they perceive that these identities can be blended, overlapped, and coexist harmoniously (Nguyen and Benet-Martínez, 2013). Rural and urban identities can be integrated because they satisfy varying needs of different life periods. For example, with regard to urban identity, migrant workers can identify with urban life to enhance their personal or career development, gain financially, and escape unfavorable rural living conditions (Meijering and van Hoven, 2003). Furthermore, the economic disparity in China encourages migrant workers to foster an urban identity to render their lives meaningful (i.e., better prospects for themselves and their children) and enhance self-esteem (i.e., expanding their urban social network and acquiring new knowledge and skills to enable them to outperform their hometown counterparts) (Wong, 2011). Meanwhile, they maintain their rural identity, stay connected with their past, and remain connected with family and friends, thereby offering self-consistency and a sense of belonging. We expect dual-identity holders to have varying organizational experiences when compared with non-dual-identity holders.

#### Dual-Identity Holders and Emotional Exhaustion: The Existential Approach to Burnout

We use the existential approach to burnout and the JD-R model to develop our hypotheses concerning the relationships of dual-identity holders, perceptions of the organization and job, and emotional exhaustion. On the basis of our approach, dual-identity holders access the objective world and deal with uncertainty differently. Thus, they acquire varying evaluative ideas regarding reality, and they experience different levels of emotional burnout.

##### The existential approach to burnout

The existential approach to burnout (Pines and Maslach, 1978; Pines, 1993) highlights two discrepancies that threaten to generate burnout. First is *life uncertainty*. That is to say, objective life experiences sometimes deviate from the ideal life, leading to feelings of emptiness and meaninglessness and energy consumption. Second is *self-concept uncertainty*. In other words, personal experience may deviate from the ideal/ought self and lead to guilt and shame, thereby depleting personal strength (Crane et al., 2008).

People may experience distant burnout as they are not always conscious of these discrepancies. Identities, which describe self-concepts (e.g., questions about who am I) and are used to interpret the environment (e.g., question about what the world is like), enable people to become aware of distant burnout threats and further proximal anxiety through interactions with specific social contexts. Empirical studies of this approach have usually been identity-specific, assuming that identity plays a defining role in influencing emotions, such as with the burnout experiences of women (Grossi et al., 2003) security guards (Rosenbloom et al., 2016) and professionals (Etzion and Pines, 1986; Schaufeli et al., 1993).

In the context of workplace, the JD-R model has become one of the most popular frameworks explaining the relationships between work environment and employee burnout (Demerouti et al., 2001; Bakker et al., 2004). The central idea of the JD-R model is that working conditions can generally be categorized as job demands or job resources, which have influences on employee well-being (Bakker and Demerouti, 2017). Job resources means “physical, social, or organizational aspects of the job that may be functional in achieving work goals, reduce job demands, and its related costs or stimulate personal growth and development” (Demerouti et al., 2001, p. 501). On the contrary, job demands refers to “physical, social, or organizational aspects of the job that require sustained physical or mental efforts” (Demerouti et al., 2001, p. 501). Emotion exhaustion can be seen as a consequence of extended exposure to specific job demands. High job demands increase the risk for burnout, while job resources play a motivating role that reduces exhaustion and stimulate engagement. In the following, it is argued, compared to the non-dual-identity holders, dual identity holders have different work perceptions that embody different types of job resources and demands and in turn affect migrant workers’ levels of burnout.

##### Dual-identity holders, the perception of internal CSR efforts, and emotional exhaustion

Employees make sense of their organizations on the basis of their experiences with company practices, policies, procedures, and rewards (Schneider et al., 2000; Bowen and Ostroff, 2004). They pay attention to their employers’ *internal CSR efforts* that include fair treatment; developmental opportunities through training (Peterson, 2004; Brammer et al., 2007; Flammer and Luo, 2017) and ethical practices, procedures, and values (Schwepker, 2001). Migrant workers attempt to interpret these practices in order to reduce work uncertainty because they may ensure that, to a great extent, employees receive the rewards they expect (Barnett and Vaicys, 2000). Migrant workers, who are more likely to suffer unfair treatment, are perhaps particularly aware of internal CSR. During the past two decades, although migrant workers have increasingly gained awareness of their social rights, these rights are still substantially infringed (Frenkel and Yu, 2015). Those workers may unwillingly experience unethical treatment, such as specific identification-card checks, hazardous work environments, unfair interpersonal interactions, and delayed salary payments (Wong, 2011).

Compared with non-dual-identity holders, dual-identity holders are more likely to perceive positive CSR treatment and evaluate organizations positively because of they owning different perspectives. Identities provide guidelines to filter and organize received information in a self-referent manner (Rogers et al., 1977). Dual-identity holders can shift their locus of identity between groups and thus the environment becomes less threatening (Roccas and Brewer, 2002). Their acceptance of two identities allows them to draw on the values of one group when they subconsciously sense threats to the other (Mok and Morris, 2010). For example, when they experience or see seemingly injust interapersonal interaction or working in a stick condition such as ID card check, dual-identity holders are often more tolerant and assume a more positive perspective toward organizational practices (Brewer and Pierce, 2005; Roccas and Amit, 2011). In addition, cross-cultural studies show that people who connect their culturally discrepant identities are more tolerant to interpersonal encounters (Huff et al., 2017) therefore they are more likely to have greater interpersonal (i.e., with migrant or local employees) support, which makes them see the environment in a more supportive manner. This leads us to our first hypothesis:

***H1:*** *Compared with non-dual-identity holders, dual-identity holders will have higher perceptions of internal CSR efforts.*

As noted earlier, the JD–R model explains how employees’ perceptions of emotional exhaustion are determined by the levels of resources at their disposal (Demerouti et al., 2001). When migrant workers see organizational with high internal CSR, they are likely to have more financial resources which are largely determined by the reward and compensation system, and emotional and information resources as a result of strengthened employee communications. When they are work in a supportive environment where safety is ensured, they may acquire deep insights into the inner workings of personnel, procedures, and resources. Thus, they are able to develop a more well-rounded view of their work and detect/explore more opportunities. This offers them the awareness of, and access to organizational resources to achieve personal success. In short, internal CSR provides resources that motivate migrant workers, thereby decreasing their emotional exhaustion. However, the perception that their employer is unethical, unfair, or unsupportive might make migrant workers feel emotional exhaustion because of the discrepancies between their life expectations and their workplace actualities (Chi and Liang, 2013). The lack of job resources and negative ethical evaluations of organizations have been known to be linked to emotional exhaustion (Bakker et al., 2004; Mulki et al., 2008; Fernet et al., 2012; Bennett et al., 2018) and workplace stress (Schwepker and Hartline, 2005). Thus, we hypothesize that,

***H2:*** *Compared with non-dual-identity holders, dual-identity holders will have lower emotional exhaustion as a result of higher perceptions of internal CSR efforts.*

##### Dual-identity holders, the perception of job complexity, and emotional exhaustion

Employees perceive job complexity when they believe that “the tasks on a job are complex and difficult to perform… require the use of numerous high-level skills and are more mentally demanding and challenging” (Morgeson and Humphrey, 2006, p. 1323). Complex jobs require the mobilization of various resources; the ability to consider various work plans and procedures; the utilization of important knowledge, skills, and abilities (KSAs); and collaboration with internal and external stakeholders (Man and Lam, 2003; Zacher and Frese, 2011).

When compared with non-dual-identity holders, dual-identity holders are more likely to perceive their jobs as being complex because they interact with more people, know more about jobs, deal with more information/collaboration, and draw feedback from multiple sources. They tend to be more experimental in view of their investing more time in KSAs to achieve work goals, which may lead to increased role and work uncertainty (Rizzo et al., 1970). Although they have multicultural schemas to understand their work, they may encounter difficulties in being recognized as being equal to locals, and they suffer when negotiating and balancing resources and interests with either group. Consequently, they always face challenges reconciling potentially conflicting identities (Ellemers, 2012). This brings us to the third hypothesis:

***H3:*** *Compared with non-dual-identity holders, dual-identity holders will have higher perceptions of job complexity.*

Although job complexity positively affects work motivation, satisfaction, and performance (Hackman and Oldham, 1976; Frese, 1982; Morgeson and Humphrey, 2006), we contend that it also leads to higher uncertainty and discrepancy between ideals and reality. Employees may suffer burnout when they feel overextended at their jobs (Maslach and Jackson, 1981). Migrant workers’ perceptions of job complexity include typical job demands such as role conflict, work pressures, and high workload that involve sustained physical and psychological efforts. According to the JD–R model, these demands in turn will deplete mental resources and impair health (Demerouti et al., 2001). Thus, job demands are positively linked to emotional exhaustion (Bakker et al., 2004; Fernet et al., 2012) and we propose the following hypothesis:

***H4:*** *Compared with non-dual-identity holders, dual-identity holders will have higher emotional exhaustion as a result of higher perceptions of job complexity.*

### The Moderating Effects of HRM Strength

Effective HRM practices send signals to employees on the organizational values, goals, and expectations codified and adhered to by the company, through extensive training programs, reward systems, and promotion criteria (Bowen and Ostroff, 2004; Li et al., 2011). Strong HRM systems have three features: distinctive and clear HR messages defined by the organizations; consistent HR messages that are signaled through different practices across time and contexts; and coherent HR implementation processes within key implementers, including HR and line managers (Sanders et al., 2014). Strong HRM systems ensure that employees understand connections between their behavior/performance and rewards and that they understand how working conditions and work relationships are organized to achieve business goals. In contrast, weak HRM systems evoke uncertainty regarding connections among job requirements, performance, organizational policies, and procedures.

We contend that strong HRM systems can weaken the differences between dual- and non-dual-identity migrants because strong systems ensure predictability of organizational treatment, rewards, behavior/performance, and job expectations. Moreover, strong systems reduce opportunities to explore different interpretations of organizations and methods of work. Therefore, we arrive at the following hypotheses:

***H5:*** *Compared with non-dual-identity holders, dual-identity holders are more likely to have higher perceptions of internal CSR efforts when HRM strength is low.*

***H6:*** *Compared with non-dual-identity holders, dual-identity holders are more likely to have higher perceptions of job complexity when HRM strength is low.*

## Method

### Participants and Procedures

Our sample included 1,985 migrant workers at 141 firms in Guangdong province, which is one of the biggest gathering places for migrant workers in China, and therefore is a representative and appropriate place to conduct this research. We firstly selected and contacted 180 firms from 1,000 firms on a list provided by the local governments based on their availability to get the supports from the firms. These firms voluntarily joined our research without any coersion and a total of 158 firms (87.78%) agreed to participate finally. The sampled firms included private firms, state-owned firms, and foreign-invested firms, and covered in both manufacturing industry and the service industry. They were located in different towns and cities in Guangdong province and were randomly selected based on the locations. Thus, although our sample may not be best representative, we have tried our best to control the sampling process and the sample used was not significantly biased. Consequently, our results could be generalized to most migrant workers in China.

We distributed manager questionnaires to the HR managers and employee questionnaires to each employee. We excluded 17 firms that provided low response rates or invalid data. The excluded firms were not significantly different from the remaining 141 sample firms with respect to basic firm information, such as industry (*t* = 0.20, *p* > 0.10) and firm size (*t* = 0.55, *p* > 0.10). The remaining firms were mostly from the manufacturing industry (*Mean* = 0.87, *SD* = 0.34), with an average of 1,538 employees (*Mean* = 1,537.98, *SD* = 2,363.93).

Employee questionnaires were distributed to 10–20 randomly selected employees at each firm. We received 1,985 valid responses accounting for about 14 valid respondents in each firm (*Mean* = 14.08, *SD* = 4.82). Men (coded 1) and women (coded 0) were equally distributed (*Mean* = 0.47, *SD* = 0.50). Their age averaged 24.98 years (*SD* = 5.62). They worked 8.98 hours daily (*SD* = 1.49). Most worked on an assembly line; about 15% had technical jobs (*Mean* = 0.15, *SD* = 0.35).

### Measures

All measures were adapted from validated scales. To ensure that the scales were valid in our research context and that migrant workers could easily understand each item, we followed translation/back-translation procedures to convert the English scales into Chinese (Brislin, 1980) and conducted 20 preliminary interviews with migrant workers before finalizing and distributing the questionnaires.

#### Dual-Identity Holders

Dual-identity holders are individuals who have high levels of urban and rural identity simultaneously. Urban identity and rural identity both were measured by five-item, five-point scales (1 = *completely disagree*, 5 = *completely agree*) adapted from Chinese migrant worker research (Guo and Li, 2009). An example of an urban identity item reads, “I consider Guangdong to be my second hometown” (α = 0.71), and a rural identity item reads, “I am still a rural person, even if have stayed in Guangdong for a long period” (α = 0.74). The full measures are listed in the Appendix. We used the median-split method (Tsui et al., 1997) to identify the dual-identity holders whose scores of both urban and rural identities were above the median. We coded the dual-identity holders as 1 and the other migrant workers as 0.

#### Perception of Internal CSR Efforts

Perceptions of internal CSR efforts were measured using a six-item scale (Li et al., 2012) based on the Social Accountability 8000 (SA8000) scale. Although CSR concerns multiple stakeholders, this scale specifically emphasizes labor protection. We consider the scale to be a proper measurement because our research question is related to employee perception of the workplace, and the preliminary interviews indicated SA8000 to be a well-accepted standard to evaluate internal CSR among sample firms. In particular, we asked respondents to evaluate whether the statements fit their actual situations, from 1 = *completely unfit* to 5 = *completely fit*. Examples include, “our firm has tried to make the workplace safe” and “our firm has tried to make the workplace comfortable” (α = 0.89). The full measure is listed in the Appendix.

#### Perception of Job Complexity

Perceptions of job complexity were measured using a three-item scale (Shaw and Gupta, 2004). Respondents indicated their agreement (1 = *strongly disagree* to 5 = *strongly agree*) with statements such as “my job is very complex” (α = 0.76).

#### Emotional Exhaustion

Emotional exhaustion was measured using a three-item scale (Karasek, 1979). Respondents indicated their agreement (1 = *strongly disagree* to 5 = *strongly agree*) with statements such as “I am continually tired during the day” (α = 0.83).

#### HRM Strength

Following Li et al. (2011), HRM strength was measured as the inverse standard deviation of employee perception of HRM at the unit level, using a seven-point scale developed by Delery and Doty (1996) and adapted to Chinese research (Zhang and Li, 2009). The profit-sharing dimension was excluded because it was irrelevant for a study of migrant workers. The measure includes six dimensions: four items for training (e.g., “extensive training programs are provided for individuals in this job”); four items for participation (e.g., “employees in this job are allowed to make many decisions”); four items for job description (e.g., “the duties of this job are clearly defined”); three items for internal career opportunity (e.g., “employees in this job who desire promotion have more than one potential position they could be promoted to”); three items for employment security (e.g., “employees in this job can expect to stay in the organization for as long as they wish”); and two items for result-oriented performance appraisals (e.g., “performance appraisals are based on objective, quantifiable results”). The total of 20 items had an overall alpha coefficient of 0.95.

#### Control Variables

We controlled for demographic information (e.g., age and gender) because younger employees (Brewer and Shapard, 2004) and women tended to indicate slightly more emotional exhaustion (Purvanova and Muros, 2010). Working time was also a key variable that led to emotional exhaustion. Therefore, we controlled for age, gender, and daily working hours when emotional exhaustion was the dependent variable. Demographic information can also influence information processing (Arcand and Nantel, 2012) in such a way that migrant workers of different ages or genders would have different perceptions of internal CSR efforts and job complexity. Perceptions of job complexity also depend on objective characteristics of the job, employee cognitive level, and employee job proficiency (Ganzach and Pazy, 2001). Therefore, we controlled for age and gender when the perception of internal CSR efforts was the dependent variable, and we further controlled for job position, education level, and job performance when the perception of job complexity was the dependent variable. Technical jobs were coded 1; non-technical jobs were coded 0. Education levels were measured as primary-school-or-below, junior-high-school, high-school, and college-or-above. Job performance was measured using a self-reported four-point scale with the following item: “I meet performance expectations” (Lepine and Van Dyne, 1998). Cronbach’s alpha was 0.84. We also controlled for basic firm information provided by the HR managers, including HR practices, industry, and firm size to control for between-firm noises. The manufacturing industry was coded as 1; others were coded as 0. Firm size was measured as the natural logarithm of the number of employees. HR practices were measured by the same scale that was previously used to calculate HRM strength.

### Analytic Strategy

Our research design included two levels, into which employees were nested within organizations, and the moderator (*HRM strength*) was a level-2 variable. Therefore, we used the multilevel path-analytical model involving a level-2 moderator in Mplus 7.4 (Muthen and Muthen, 1998) to test our hypotheses. The level-1 variable included dual-identity, perceptions of internal CSR efforts, perceptions of job complexity, and emotional exhaustion. The level-2 variable included HRM strength (Figure 1). Following Koopman et al. (2016) we used random slopes for level-1 variables and allowed for two mediators to covary: perceptions of internal CSR efforts and those of job complexity. Control variables were modeled with fixed slopes. For the mediation hypotheses, we conducted Monte Carlo simulations with 20,000 replications and computed 95% confidence intervals (CI) to test the significance of the indirect effects of dual-identity on emotional exhaustion in dual-identity holders via perceptions of internal CSR efforts and those of job complexity (Selig and Preacher, 2008). We also centered the predictors to alleviate potential multicollinearity (Hofmann and Gavin, 1998).

## Results

### Descriptive Results

We first performed Harman (1976) one-single-factor test to determine whether one general factor explained the most variance, considering that we collected individual-level variables from a single source. Thus, common-method variance potentially prevented the drawing of convincing conclusions. The model included all the scale-measured variables: rural identity, urban identity, perceptions of HR practices, perceptions of CSR and job complexity, and emotional exhaustion. The one-factor confirmatory factor analysis results suggested a poor fit with the data (χ^2^ = 14,605.42, d.f. = 819; CFI = 0.57; RMSEA = 0.09; SRMR = 0.09) (Hu and Bentler, 1995). Therefore, common-method variance was not a major issue.

Table 1 shows the descriptive statistics and correlations matrix for variables at both levels. Dual-identity holders constituted approximately 23% of the sample (*Mean* = 0.23, *SD* = 0.42). They were comparatively more likely to perceive higher levels of internal CSR efforts (*r* = 0.18, *p* < 0.01) and job complexity (*r* = 0.13, *p* < 0.01) but were not more likely to feel exhausted (*r* = 0.02, *p* > 0.10). Older migrant workers were more likely to perceive higher internal CSR efforts (*r* = 0.09, *p* < 0.01) and job complexity (*r* = 0.09, *p* < 0.01). Women were more likely to perceive lower levels of internal CSR efforts (*r* = −0.06, *p* < 0.05) but were less likely to perceive higher job complexity (*r* = 0.11, *p* < 0.01). Employees who perceived high internal CSR efforts were less likely to feel exhausted *(r* = −0.23, *p* < 0.01); however, those who perceived higher job complexity were more likely to feel exhausted (*r* = 0.18, *p* < 0.01).

**TABLE 1:** Means, standard deviations, and inter-correlations.

**Variable Names**		**Mean**	***SD***	**1**	**2**	**3**	**4**	**5**	**6**	**7**	**8**	**9**
**Level 1**												
1	Emotional exhaustion	2.85	1.09									
2	Perception of internal CSR efforts	3.46	0.86	−0.23**								
3	Perception of job complexity	2.79	0.89	0.18**	0.11**							
4	Dual-identity holders	0.23	0.42	0.02	0.18**	0.13**						
5	Gender	0.47	0.50	0.01	−0.06*	0.11**	−0.05*					
6	Age	24.98	5.62	−0.13**	0.09**	0.09**	0.01	0.15**				
7	Education	2.66	0.70	–0.03	–0.02	0.03	–0.04	0.04	−0.14**			
8	Job position	0.15	0.35	0.02	–0.01	0.19**	–0.01	0.26**	0.07**	0.09**		
9	Performance	3.87	0.71	0.02	0.18**	0.14**	0.11**	–0.00	0.06**	0.14**	0.04	
10	Working hours	8.98	1.49	0.23**	−0.17**	0.08**	–0.01	0.07**	−0.06**	−0.12**	0.04	–0.03
**Level 2**												
1	HRM strength	1.54	0.31									
2	Firm HR practices	5.34	1.07	0.01								
3	Industry	0.87	0.34	0.14	0.04							
4	Firm size	6.59	1.21	0.17*	0.24**	0.39**						

Gender: 1 = man, 0 = woman; Job position: 1 = technical job, 0 = other jobs; Industry: 1 = manufacturing industry, 0 = others; firm size is measured as natural logarithm of the number of the employees; *p < 0.05; *p < 0.01.

### Test of Hypotheses

Before testing the hypotheses, we first estimated null models in which no predictors were specified at either level to examine whether significant between-group variance occurred in the areas of emotional exhaustion and perceptions of internal CSR efforts and job complexity. Results indicated significant between-group variance for the dependent variables: *emotional exhaustion*, σ^2^ = 1.03, τ^00^ = 0.08, *x*^2^ = 120.11, *p* < 0.01; *perceptions of internal CSR efforts*, σ^2^ = 0.65, τ^00^ = 0.08, *x*^2^ = 25.53, *p* < 0.01; and *perceptions of job complexity*, σ^2^ = 0.70, τ^00^ = 0.04, *x*^2^ = 88.18, *p* < 0.01.

**FIGURE 2:**
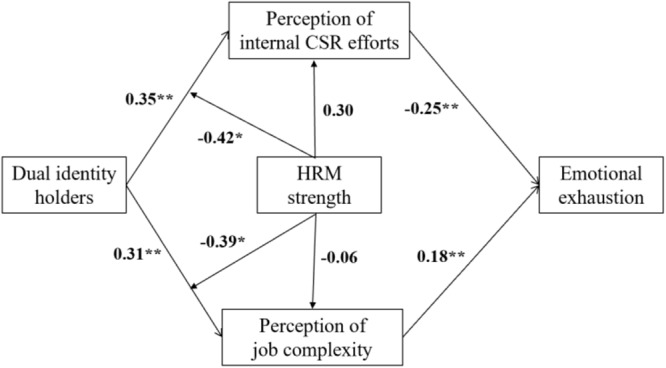
Results testing all hypotheses.

As Figure 2 shows, dual-identity holder was positively associated with perceptions of internal CSR efforts (γ = 0.35, *p* < 0.01), supporting Hypothesis 1. Hypothesis 2, which predicted the perception of internal CSR efforts as mediating the relationship between the dual-identity holder and emotional exhaustion, was supported because as Hypothesis 1 suggested, dual-identity holder was positively associated with the perception of internal CSR efforts. Furthermore, while controlling for the dual-identity holders, the perception of internal CSR efforts was negatively associated with emotional exhaustion (γ = −0.25, *p* < 0.01), and the 95% CI for the indirect effect did not include 0 (indirect effect = −0.09, 95% CI = [−0.12, −0.06].

Similarly, Hypothesis 3 was supported. Dual-identity was positively associated with the perception of job complexity (γ = 0.31, *p* < 0.01). Hypothesis 4 was supported because as suggested by Hypothesis 3, dual-identity holders were was positively associated with the perception of job complexity. Controlling for the dual-identity holders, the perception of job complexity was positively associated with emotional exhaustion (γ = 0.18, *p* < 0.01). The 95% CI for this indirect effect did not include zero (indirect effect = 0.06, 95% CI = [0.04, 0.09].

**FIGURE 3:**
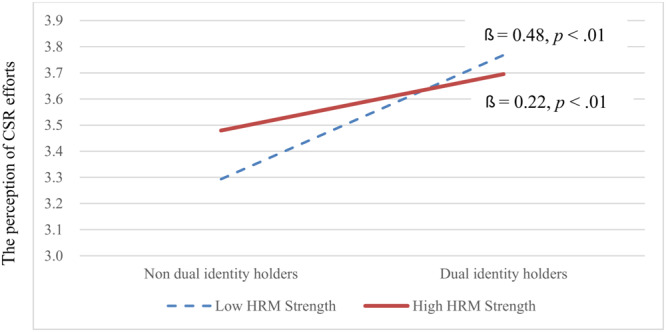
Cross-level moderating effect of HRM strength on the relationship between dual-identity holders and the perception of internal CSR efforts.

**FIGURE 4:**
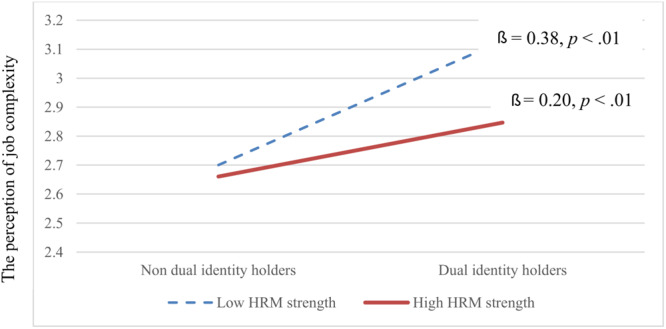
Cross-level moderating effect of HRM strength on the relationship between dual-identity holders and the perception of job complexity.

Hypothesis 5, regarding cross-level moderation, was supported (γ = −0.42, *p* < 0.05). Under weak HRM, dual-identity holders were more likely to have comparatively higher perceptions of internal CSR efforts (Figure 3). In particular, dual-identity holder was positively associated with perceptions of internal CSR efforts under low (*β* = 0.48, *p* < 0.01) and high (*β* = 0.22, *p* < 0.01) HRM strengths. However, the relationship was significantly stronger under weak HRM. Similarly, Hypothesis 6 was supported (γ = −0.39, *p* < 0.05). Under weak HRM, dual-identity holders were more likely to have comparatively higher perceptions of job complexity (Figure 4). Particularly, dual-identity holders were positively associated with perceptions of job complexity under low (*β* = 0.38, *p* < 0.01) and high (*β* = 0.20, *p* < 0.01) HRM strength. However, the relationship was stronger under weak HRM.

### Supplemental Analyses

We also examined the moderated mediation hypotheses in terms of whether HRM strength moderated the mediated relationships in Hypotheses 2 and 4. As Table 2 shows, dual-identity holder was significantly associated with emotional exhaustion via the perception of internal CSR efforts under low (indirect effect = −0.12, 95% CI = [−0.17, −0.06]) and high HRM strengths (indirect effect = −0.06, 95% CI = [−0.10, −0.02]). The difference was significant (diff = 0.06, 95% CI = [0.01, 0.11]). Dual-identity was significantly associated with emotional exhaustion via the perception of job complexity under low (indirect effect = 0.08, 95% CI = [0.04, 0.11]) and high HRM strength (indirect effect = 0.04, 95% CI = [0.01, 0.06]), and the difference was marginally significant (diff = −0.04, 95% CI = [−0.08, 0.00], 90% CI = [−0.08, −0.01]).

**TABLE 2:** Supplemental moderated mediation results.

	**High**		**Lower**		**Difference**	
**Mediators**	**Lower**	**Upper**	**Lower**	**Upper**	**Lower**	**High**
Internal CSR efforts	–0.10	–0.02	–0.17	–0.06	0.01	0.11
Job complexity	0.01	0.06	0.04	0.11	–0.08	0.00

## Discussion

This study investigated how dual-identity holders can hold different perceptions toward their organizations and jobs, which further leads to different levels of emotional exhaustion. Using a sample of 1,985 migrant workers from 141 organizations, we found two parallel paths to explain whether dual-identity holders were more or less likely to feel emotional exhaustion. First, the motivational path indicated that dual-identity migrant workers experienced less emotional exhaustion because of their higher perceptions of internal CSR efforts. Second, the health-impairment path indicates that dual-identity migrant workers experience more emotional exhaustion because of their higher perceptions of job complexity. Furthermore, we found that HRM strength indicated a more predictable workplace and thus weakened the differences in work perceptions between dual-identity holders and non-dual-identity holders.

### Theoretical Implications

Our findings provide preliminary evidence that dual-identity holders may differ from other migrant workers in terms of workplace experiences. Although scholars such as Ang et al. (2003) and Nielsen et al. (2011) have demonstrated that migrant workers, differentiated on the basis of nationality and *hukou* status, harbored different work perceptions and reported well-being, our study was among the first to use two dimensions of identity (i.e., home community vs. host community) to differentiate migrant workers and further examine the effects of identities in a more refined manner. Our findings align with SIC, and biculturalism literature suggesting that people who embrace multiple identities are more likely to have positive perceptions, interact with different people, and seek more options and resources to achieve their goals. Future research may extend this logic and line of enquiry by examining more work-related perceptions, such as organizational justice and support, to enable the establishment of systematic comparisons between dual-identity holders and their migrant counterparts. Furthermore, our studies focus on migrant workers from rurals to urban areas in China, which means, they are in general receive low or moderate levels of education, and unskilled in workplace (Syed, 2008). Future studies may also consider skill levels and cultural or value orientations as potential moderators when examining migrant workers’ work-related perceptions.

Our study adds relevant understanding to the academic discussions of workplace interpretations (Weick, 1995). We integrated the rationalizing literature with SIC, identity integration/biculturalism, and acculturation to better understand how migrant workers, particularly dual-identity holders, acclimatize to, and come to form a perception of, the workplace. Specifically, dual-identity holders appear to be experimental in reconnoitering and coming to grips with the environment (Good et al., 2016). They are less likely to use the past-experience-based ego to interpret external stimuli, thus being less threatened by negative workplaces (Glomb et al., 2011). Future research should explore more perspectives to comprehensively understand how migrant workers interpret the workplace (Lucas et al., 2013; Thomson and Jones, 2017).

Our findings are consistent with the JD–R literature. We identify that perceptions of job complexity can generate burnout although job complexity has been shown to indicate opportunity, thereby increasing personal initiative (Fay and Kamps, 2006; Zacher and Frese, 2011). These mixed findings indicate other moderators in the job complexity–burnout relationship. We suggest that other moderators might include inferior status, cultural orientation regarding self-enhancement values, and coordination capabilities (Marques-Quinteiro et al., 2013). Therefore, further job complexity research is needed.

We showed that HRM strength greatly affects migrant workers’ workplace perceptions. When HR agents consistently and coherently convey messages regarding HRM systems, employees form shared understandings regarding organizational values, expectations, and rewards (Li et al., 2011). Consequently, they showed fewer exploratory or opportunistic interactions and behaviors, and their job-related perceptions were more closely aligned with organizational values. To expand upon this idea, future research should test more individual-level relationships with HRM strength as a conditional variable defining organizational predictability.

### Practical Implications

Our study has practical implications for migrant workers. That is to say, they must realize the significance of managing their social identities. Embracing multiple identities can have advantages in widening perceptions, expanding contacts, and finding opportunities and resources to achieve personal goals. Key disadvantages include lost time, the energy expended to experiment with different options, and the energy required in order to deal with potential conflicts. These motivational and health-impairment elements coexist every workday and can deplete energy. Workers must balance their personal resources with their self-concepts regarding the ideal and the actual self and the organizational environment. To balance these self-concepts, migrant workers are advised to learn from dialectical thinking style, which employs a unique perspective on change (Peng and Nisbett, 1999). Dialectical thinkers see things in a dynamic world and are always changing, which allows them to see contractionary propositions can coexist in a harmonious way. This approach may reduce dual-identity holders’ exhaustion when experiencing conflicts. Further, they can also be active to contact friends and family members in and outside the firms to gain social support, which is an important source for psychological resources to reduce work-related stress.

Our study also has practical implications for managers and organizations. That is to say, they should realize that migrant workers will have varying perceptions of internal CSR efforts and job complexity levels. Policies and practices must be explicated clearly and consistently to ensure employee understanding of organizational goals and the means to achieve these goals. Employees should be shown the manner in which to link organizational goals to their personal goals. Diversity programs should be proactively introduced to legitimize and support the coexistence of multiple identities (Cole and Salimath, 2013) and to make employees feel supported, included, and hopeful.

### Limitations

This study has three major limitations. First, we conducted a one-time survey. Thus, causality may be an issue. As discussed, reverse causality is unlikely for several reasons. First, identity complexity is determined by value orientation and fundamental personal belief rather than workplace perceptions and emotional statuses. Second, the Hackman one-factor test suggested a low fit between our data and the one-factor measurement structure. Third, we based our theory on an existential approach that called for burnout to be influenced by occasional subconscious identities and cognitive representations of the world. In addition, consistent with the JD–R model, we concluded that reverse relationships between emotion exhaustion and work perceptions were unlikely.

The second limitation concerns the measure of dual-identity holders. The literature has provided several measures. In the acculturation literature, measures focused on migrants’ attitudes, motivations, and behaviors toward individuals with home or host origins (Berry, 1990, 2009). In the biculturalism literature, identity integration measured the focus on the blendedness and harmony of two cultures (Benet-Martínez et al., 2002; Ng et al., 2010; Huff et al., 2017). However, our conceptualization of dual-identity was consistent with SIC theory. Dual-identity holders integrate different identities and switch from one to another in different contexts. Future research should seek out a better proxy variable or measures to align with SIC theory.

The third limitation is related to the sample. Migrant workers in China have their own social and cultural backgrounds. Nevertheless, just as with migrant workers everywhere, they have inferior statuses both inside and outside the workplace (Syed, 2008; Ciupijus, 2010). Future studies are in order for exploring whether the theoretical model can be supported in other contexts.

## Conclusion

In this article, we provided insights into the work experiences of migrant workers. We surveyed 1,985 employees at 141 firms and found that migrant workers experienced emotional exhaustion upon perceiving that their employers failed to provide CSR support and that their jobs were highly complex. Dual-identity holders, in contrast, were given to perceive stronger CSR support and thus experienced less emotional exhaustion; however, their health was sometimes impaired by higher perceptions of job complexity. Strong HRM services weakened those relationships. These findings are expected to have far-reaching implications for migrant workers and for highly diverse organizations.

## Data Availability Statement

The datasets generated for this study are available on request to the corresponding author.

## Ethics Statement

The studies involving human participants were reviewed and approved by the Guanghua School of Management, Peking University. Written informed consent for participation was not required for this study in accordance with the national legislation and the institutional requirements.

## Author Contributions

XL, HZ, and JZ designed the study together and revised the draft together. HZ and JZ collected the data. XL drafted the theory. HZ drafted the method and results.

## Conflict of Interest

The authors declare that the research was conducted in the absence of any commercial or financial relationships that could be construed as a potential conflict of interest.

## Appendix

### Urban Identity, Rural Identity, and Perception of Internal CSR Efforts Scale Items

**Table:**

**Urban identity**
(1) I consider Guangdong to be my second hometown. (2) I have gotten used to urban life and feel uncomfortable when I return to the rural area I come from. (3) I would strongly regret leaving the city if I had to. (4) I am part of the community here. (5) I feel that I am connected with the city.
**Rural identity**
(1) I am still a rural person even if have stayed in Guangdong for a long period. (2) I find it very difficult to leave my hometown for Guangdong. (3) I often miss my previous life in my hometown. (4) I often call people in my hometown to learn about what happens there. (5) I have many close friends in my hometown.
**Perception of internal CSR efforts**
(1) Our firm has tried to make the workplace safe (2) Our firm has tried to make the workplace comfortable. (3) Our firm has tried to make the reward and punishment system reasonable. (4) Our firm has tried to improve compensation. (5) Our firm has tried to reduce labor intensity. (6) Our firm has tried to strengthen communications with employees.
